# Combination model for freshness prediction of pork using VIS/NIR hyperspectral imaging with chemometrics

**DOI:** 10.5713/ab.24.0255

**Published:** 2024-08-26

**Authors:** Minwoo Choi, Hye-Jin Kim, Azfar Ismail, Hyun-Jun Kim, Heesang Hong, Ghiseok Kim, Cheorun Jo

**Affiliations:** 1 Department of Agricultural Biotechnology and Center for Food and Bioconvergence, Seoul National University, Seoul 08826, Korea; 2 Department of Aquaculture, Faculty of Agriculture, Universiti Putra Malaysia, Selangor 43400, Malaysia; 3 Department of Biosystems Engineering, Seoul National University, Seoul 08826, Korea; 4 Research Institute of Agriculture and Life Sciences, Seoul National University, Seoul 08826, Korea; 5 Institute of Green Bio Science and Technology, Seoul National University, Pyeongchang 25354, Korea

**Keywords:** Combination Model, Freshness, Hyperspectral Imaging, Metabolites, Pork

## Abstract

**Objective:**

This study aimed to develop an enhanced model for predicting pork freshness by integrating hyperspectral imaging (HSI) and chemometric analysis

**Methods:**

A total of 30 *Longissimus thoracis* samples from three sows were stored under vacuum conditions at 4°C±2°C for 27 days to acquire data. The freshness prediction model for pork loin employed partial least squares regression (PLSR) with Monte Carlo data augmentation. Total bacterial count (TBC) and volatile basic nitrogen (VBN), which exhibited increases correlating with metabolite changes during storage, were designated as freshness indicators. Metabolic contents of the sample were quantified using nuclear magnetic resonance.

**Results:**

A total of 64 metabolites were identified, with 34 and 35 showing high correlations with TBC and VBN, respectively. Lysine and malate for TBC (R^2^ = 0.886) and methionine and niacinamide for VBN (R^2^ = 0.909) were identified as the main metabolites in each indicator by Model 1. Model 2 predicted main metabolites using HSI spectral data. Model 3, which predicted freshness indicators with HSI spectral data, demonstrated high prediction coefficients; TBC R^2^_p_ = 0.7220 and VBN R^2^_p_ = 0.8392. Furthermore, the combination model (Model 4), utilizing HSI spectral data and predicted metabolites from Model 2 to predict freshness indicators, improved the prediction coefficients compared to Model 3; TBC R^2^_p_ = 0.7583 and VBN R^2^_p_ = 0.8441.

**Conclusion:**

Combining HSI spectral data with metabolites correlated to the meat freshness may elucidate why certain HSI spectra indicate meat freshness and prove to be more effective in predicting the freshness state of pork loin compared to using only HSI spectral data.

## INTRODUCTION

Pork is known to be susceptible to the loss of freshness and finally spoilage primarily due to its high protein content, vitamins, minerals, and other nutrients [1]. In the United States, the losses attributed to pork spoilage amount to USD 1 billion annually [2]. Since the sources of pork freshness deterioration are diverse, including meat oxidation, endogenous enzymes from the meat, and exogenous enzymes from microorganisms, preventing spoilage during storage becomes a considerable challenge [3,4]. Therefore, monitoring the freshness of pork during distribution is of paramount importance.

There are various traditional methods to measure meat freshness, such as total bacterial count (TBC), volatile basic nitrogen (VBN), pH, thiobarbituric acid reactive substances (TBARS), biogenic amine, meat color, drip loss, etc. [5,6]. Among them, we focused on TBC, VBN, pH, and meat color. Firstly, TBC represents the number of microorganisms, and a higher TBC indicates a poorer state of fresh meat [7]. The level of total VBN also increases due to the decomposition of muscle proteins into amino acids by enzymes secreted by microorganisms [8]. Then, pH changes are related to bacterial growth during spoilage, involving bacteria such as *Pseudomonas* spp. and *Brochothrix thermosphacta* [9]. Lastly, because the color of meat significantly influences consumers’ decisions when purchasing meat, discoloration in meat could be considered spoilage and used as an indicator of meat freshness [10]. Even though these methods have been widely employed to measure meat freshness, there is a growing demand for non-destructive, rapid, and accurate analytical methods due to their destructive and time-consuming nature [11].

Hyperspectral imaging (HSI) is an analytical technology that combines conventional computer imaging with spectral analysis methods for the simultaneous acquisition of spectral and spatial information in rapid and non-destructive ways [12]. Through HSI analysis, hyperspectral data containing three-dimensional information, known as a hypercube, is obtained. This hypercube encompasses both spectral information (1D) and spatial information (2D) [13]. Numerous studies have created models predicting meat freshness using HSI. Zhuang et al [14] established a partial least squares regression (PLSR) model to predict the freshness and quality traits of frozen pork using Vis/NIR HSI. Park et al [15] attempted to predict the TBARS value of beef using the line-scan HSI system. However, the explanation between meat freshness and HSI spectral data has not been fully elucidated.

Metabolites are compounds that play a role in regulating numerous biochemical pathways and are sometimes utilized as indicators of biological conditions [16]. It has been reported that metabolites in meat undergo changes during storage, impacting meat quality factors such as tenderness, taste, and aroma [11,17,18]. Dave and Ghaly [18] stated that microbial metabolites, including aldehydes, alcohols, ketones, esters, amines, organic acids, and sulfur compounds, accumulate during the storage process and contribute to meat spoilage. Therefore, understanding the metabolic changes during storage could shed light on the spoilage pattern in pork meat. Some research has utilized HSI to predict metabolites in the meat domain [19,20]. Consequently, analyzing metabolites correlated with the freshness of meat could possibly explain how HSI spectral data suggests meat freshness.

Therefore, this study aimed to develop a highly predictive model for pork freshness prediction using HSI and chemometrics analyses. To achieve this objective, the relationship between freshness and metabolites was analyzed, and a novel combination model for the freshness of pork meat was established with metabolites via HSI. This approach followed the method employed by Zuo et al [21], who developed a protein content prediction model using HSI-predicted fat content and HSI data as input variables.

## MATERIALS AND METHODS

### Sample preparation

Loin portions (*Longissimus thoracis*) from both sides of three sows were purchased from three different butcher shops. Each sow had been raised on a different farm and slaughtered two days before obtaining the loin portions. Physicochemical, chemometric analysis, and HSI spectral data collection of each side of the loin are shown in the supplementary material (Supplementary Figure S1). After removing the excess fat from the loin, it was cut to approximately 11-cm thickness and vacuum-packaged (HFV-600L; Hankook Fujee Machinery Co., Ltd., Hwaseong, Korea) using a polyethylene/nylon bag (with an oxygen permeability of 22.5 mL/m^2^/24 h atm at 60% relative humidity (RH)/25°C and a water vapor permeability of 4.7 g/m^2^/24 h at 100% RH/25°C). A total of 30 loin cuts (5 days× 3 animals×2 sides) were stored at 4°C±2°C for 1, 4, 13, 20, and 27 days and were used for analysis.

### Microbial and physicochemical characteristics of the meat

#### Total bacterial count

TBC was conducted following the procedure outlined by Ismail et al [22]. Ten grams of samples were transferred to a sterile bag containing 90 mL of 0.85% NaCl solution. After mixing using a BagMixer 400P (Interscience Ind., St. Nom, France), serial dilutions were performed to obtain countable concentrations. Subsequently, 100 μL aliquots of appropriate dilutions were inoculated on plate count agar (Difco Laboratories, Detroit, MI, USA), incubated at 37°C for 48 h, and then colonies were counted. Finally, the results were expressed as log CFU/g.

#### Volatile basic nitrogen

The method for measuring the VBN content of pork loin followed that outlined by Ismail et al [22]. Three grams of sample were homogenized with 27 mL of distilled water using a homogenizer (Ultra-Turrax T25; Ika-Werke, Staufen, Germany) at 9,500 rpm for 30 s. The homogenates were then centrifuged (Union 32R; Hanil, Incheon, Korea) at 2,265×g for 10 min and filtered using filter paper (Whatman No. 1; Whatman plc, Maidstone, UK). Subsequently, 1 mL of each sample, 50% K_2_CO_3_, and 0.01 N H_3_BO_3_, along with 100 μL of the indicator (0.066% methyl red in ethanol: 0.066% bromocresol green in ethanol, 1:1, w/v) were poured into the Conway (Sibata Ltd., Sitama, Japan). Finally, the Conway was sealed and incubated (DS-130L; Daewon Sci Co., Bucheon, Korea) at 37°C for 1 to 2 h. After incubation, color changes were observed and recorded by adding 0.01 N HCl to the center of the Conway, and VBN was calculated using the formula below:


$$\text{VBN }(\text{mg}/100\;\text{g of sample})=[0.14\times (V_{1}-V_{0})\times 10\times 100]/\text{S}$$

where *V*_1_ represents the amount of 0.01 N HCl added to the treatment, *V*_0_ represent that added to the control. S denotes the sample weight, and lastly, 0.14 indicates the quantity of volatile basic nitrogen per 1 mL of 0.01 N HCl solution.

#### pH

The pH measurement method followed the procedure outlined by Lee et al [23]. Three grams of each sample were homogenized with 27 mL of distilled water utilizing an Ultra-Turrax T25 homogenizer (Ika-Werke, Germany) at 9,600 rpm for 30 s. Subsequently, the homogenates were centrifuged (Continent 512R; Hanil, Korea) at 2,265×g for 10 min and then filtered (Whatman No. 4; Whatman plc, UK). The pH of the samples was measured by pH meter (Seven2Go; Metter-Toledo International Inc., Schwerzenbach, Switzerland) with the pH probe (InLab Expert Go-ISM; Metter-Toledo International Inc., Switzerland). After calibrating the pH meter using three buffer solutions (pH 4.0, 7.0, and 9.21) at room temperature, the pH of each filtrate was measured twice, and the average value was used as a representative replicate.

#### Meat color

The assessment of meat color was conducted using a colorimeter (CM-5; Konica Minolta Sensing Inc., Osaka, Japan) following Kim et al [24]. The observer degree was set at 10, with illuminants being D65 and C. Prior to measurement, the sample underwent a 30 min exposure to air to bloom, and the colorimeter was calibrated using a standard white plate. Meat color was measured using a 30 mm diameter plate. The results were expressed in terms of Commission Internationale d’Eclairage (CIE) L*, a*, and b*, representing lightness, redness, and yellowness, respectively. Each sample was measured five times, and the mean value was used as a singular replicate.

### Nuclear magnetic resonance

The nuclear magnetic resonance (NMR) experimental method for analyzing the metabolites followed that outlined by Lee et al [25]. First, one gram of pork loin was prepared, and 4 mL of 0.6 M perchloric acid was added. The mixture was homogenized at 16,000 rpm for 1 min using a T25 Ultra homogenizer (Ika-Werke, Germany). The homogenate was then centrifuged at 3,000×g for 20 min at 4°C using a Continent 512R centrifuge (Hanil Co., Ltd., Korea). After transferring the obtained supernatant to a new tube, the pH was adjusted to 7 with a pH meter (SevenGo; Mettler-Toledo, Schwerzenbach, Switzerland) using KOH, and centrifugation was performed again at 3,000×g for 20 min at 4°C. Afterward, the solution was filtered through Whatman No.1 filter paper (Whatman plc, UK). Subsequently, the filtrate was freeze-dried using a freezer dryer 18 (Labco Corp., Kansas City, MO, USA). The lyophilized sample was reconstituted using 1 mM 3-(trimethylsilyl) propionic acid-2,2,3,3-*d*_4_ (internal standard, TSP) in D_2_O (pH 7.4, 20 mM phosphate buffered), vortexed and stored in a water bath at 37°C for 10 min. After samples were centrifuged at 17,800×g for 20 min (HM-150IV; Hanil Co., Ltd., Korea), samples were loaded into 5 mL NMR tubes, and metabolites were analyzed by an NMR spectrometer.

The NMR data were collected at 298 K using a Bruker 850 MHz NMR spectrometer (Bruker Biospin GmbH, Baden-Wuttemberg, Germany). The standard zg30 pulse sequence was employed for the analysis of 1D ^1^H NMR in Topspin 4.2.0 (Bruker, Germany). Pulse sequences were obtained using 64 K data points, a sweep width of 17,007.803 Hz, and 128 scans and the acquisition time was 4.20 s. TSP resonance served as the reference for the chemical shifts (δ) in both qualification and quantification processes. Baseline corrections were performed manually. For peak identification, 2D NMR spectra such as correlation spectroscopy (COSY) and heteronuclear single quantum coherence (HSQC) were collected for metabolite qualification. COSY was conducted with 2 K data points in the t2 domain and 128 increments in the t1 domain with 16 scans, and the spectral width was 11 ppm. HSQC was executed with 2 K data points in the t2 domain and 256 increments in the t1 domain with 16 scans, and 223 ppm for the F1 and 11 ppm for the F2 axis, respectively. A coupling constant of 145 Hz determined the delay duration for short-range correlations. Additionally, peaks in the measured 2D spectra were identified based on the Human Metabolome Database (HMDB; hmdb.ca). Peaks identified by 2D HSQC NMR were then quantified using ^1^H NMR spectroscopy. The quantified dataset from the ^1^H NMR spectrum of each metabolite was processed using Chenomx NMR suite 10.0 (Chenomx, Inc., Edmonton, AB, Canada). A 1 mM TSP served as the internal standard for metabolite quantification. The quantification of samples was conducted with five replicates, and the unit of the metabolite concentration was expressed as mg/dL.

### Data processing and statistical analysis of quality and metabolites data

The meat quality traits (TBC, VBN, pH, L*, a*, and b*) and metabolites were analyzed in 6 replicates for each storage day, and the sample which showed the highest noise in the NMR analysis at each storage day was considered an outlier and excluded from the dataset (n = 25, 5 storage days×5 replication). The results were analyzed via a one-way analysis of variance with the generalized linear model using SAS 9.4 (SAS Institute Inc., Cary, NC, USA). The storage day was set as the only fixed effect. Tukey’s multiple tests were used to determine significant differences between sample groups at p<0.05.

Multivariate and correlation analyses were conducted using MetaboAnalyst 5.0. A hierarchical clustering heatmap was employed to identify changes in metabolite content and meat qualities over storage days. Additionally, principal component analysis (PCA) and partial least squares-discriminant analysis (PLS-DA) were performed to determine which metabolites contribute to the separation of samples based on the storage duration. Through the PLS-DA results, variable importance in projection (VIP) scores for each metabolite was obtained. VIP scores serve as indicators revealing which metabolites significantly contribute to the separation of samples. Additionally, the Pattern Hunter feature in MetaboAnalyst was utilized to generate bar graphs comparing the correlation between the metabolites and quality characteristics using the Pearson correlation coefficient. Finally, the implications of the observed high correlation were explored.

### Hyperspectral imaging system and data acquisition

Hyperspectral imaging analysis was conducted utilizing a push-broom scanner featuring an HSI-200 sensor (Korea Spectral Products, Seoul, Korea). To ensure the complete elimination of external light during image acquisition, an image acquisition system was installed in a dark condition. The HSI system was equipped with an imaging spectrometer with a resolution of 640 spectral×512 spatial, generated using an InGaAs imaging sensor covering the spectral range from the visible to short-wave near-infrared regions. Tungsten halogen lamps were employed to provide sample illumination for uniform lighting during the imaging process. Each image pixel comprised 640 wavelengths, spanning the spectrum from 278 to 1,724 nm. The pork loin was cut into one replication at 3×3×1 cm (width×length×height) sizes and a total of 100 samples (5 storage days×5 replication×4 observations) were analyzed. A Teflon whiteboard (99.99% reflectivity) was used to acquire the white reference, while the dark reference was obtained by covering the camera (0% reflectance). This step aimed to eliminate the dark current effect and minimize the impact of uneven illumination, resulting in a normalized range from 0 to 1. The normalized reflectance data were calculated using the following equation [26].


Reflectance=(S-D)/(W-D)

S represents the intensity of the sample, D denotes the intensity of the dark reference, and W signifies the intensity corresponding to the white reference. After constructing the reflectance data, wavelengths identified as outliers were considered noise and subsequently removed. Therefore, the final spectral range was from 400 to 1,600 nm.

### Modeling

The entire modeling process is succinctly outlined in Supplementary Figure S2. For modeling, all the meat quality data were matched with the HSI spectral data from the same sample. The modeling proceeded in four steps, including PLSR modeling using MATLAB (R2022b; The Mathworks Inc., Natick, MA, USA) and linear regression modeling using the SPSS program (version 26; IBM, Armonk, NY, USA). The PLSR method can maximize the correlation between the principal components extracted from the input and output by combining PCA, multiple linear regression, and canonical correlation. This method is known to effectively solve the multiplicity correlation problem of variables [14]. An imbalanced and small dataset is known to make the training process challenging in modeling [27]. To address this issue, the Monte Carlo method was utilized to augment the dataset (n = 10,000; [13]) from 100 data (5 storage days ×5 replication × 4 observations).

#### Model 1 (Metabolites to meat quality): Predicting meat quality of pork loin using metabolites

PLSR models were established to predict TBC, VBN, pH, L*, a*, and b* values using metabolites. The coefficient of determination (R^2^) and root mean squared error (RMSE) of each model were collected to evaluate the performance of the developed models. Initially, the augmented dataset (n = 10,000) was divided into calibration and prediction sets in a 7:3 ratio for model creation and validation [13]. Subsequently, PLSR models for each quality parameter were obtained using MATLAB with the PLS-Toolbox (R9.2.1; Eigenvector Inc., Wenatchee, WA, USA) [28]. The optimized latent variables (LVs) were selected using the Venetian blinds cross-validation method, employing 10 splits with 1 sample per split, based on the lowest root mean square error of cross-validation (RMSECV) values. The prediction set’s R^2^ value for each model was then compared, and the top-quality indicators with the highest R^2^ values were selected as freshness indicators. Metabolites showing a high association with the freshness indicators were identified (VIP scores>1.0).

#### Equation: Linear regression of meat freshness indicators using identified metabolites

To calculate freshness indicators (Y) with the identified metabolites (X) based on VIP scores (>1.0), linear regression models were established using the SPSS program. The stepwise method was used to input identified metabolites, and models were chosen considering the variance inflation factor (VIF) and condition index (CI) not exceeding 10 and 15, respectively [29]. When creating these equations, the unaugmented dataset (n = 25) was used. Ultimately, linear relationship equations for selected metabolites and freshness indicators were established.

#### Model 2 (HSI to metabolite): Predicting metabolites of pork loin using HSI spectral data

For making PLSR models of the selected metabolites which compose the linear equation of the freshness indicators using HSI spectral data, the augmented dataset (n = 10,000) was divided into calibration and prediction sets in a 7:3 ratio. Subsequently, to optimize model performance, different preprocessing methods for the HSI data were applied to the PLSR model, including standard normal variate (SNV), normalization, and multiplicative scatter correction (MSC). Preprocessing should be applied to reduce the noise that occurs in HSI spectral data during the measurement process [22]. All three methods were expected to reduce noise arising from spectral data. SNV is a statistical method employed to eliminate slope variation in spectra and mitigate its scatter effects [30]. Normalization is used to correct spectral noise resulting from variations in the optical path of sight [31]. Lastly, the MSC method can alleviate noise by correcting the light scattering from the different particle sizes of the sample [30]. Afterward, the best model for each metabolite was selected based on the highest R^2^ value.

#### Model 3 (HSI to freshness): Predicting meat freshness indicators of pork loin using HSI spectral data

The augmented dataset was utilized and divided into calibration and prediction sets (n = 10,000; 7:3 ratio). Subsequently, HSI data were subjected to different preprocessing methods, including SNV, normalization, and MSC. Finally, PLSR models for each freshness indicator of pork loin were developed using preprocessed HSI spectral data. The models with the highest R^2^ value were then selected as the best models for each freshness indicator.

#### Model 4 (Combination model): Predicting meat freshness indicators with both HSI spectral data and selected metabolites

To enhance model performance for predicting meat freshness indicators, the combined method was used [21]. The combined X variables comprised the predicted metabolites (n = 3,000) resulting from Model 2 and the prediction dataset of HSI spectral data (n = 3,000) from Model 2 and 3. Additionally, Y variables were defined as the prediction dataset of freshness indicators from Model 3 (n = 3,000). This combined dataset was again divided into calibration and prediction sets with a 7:3 ratio (2,100:900), applying various preprocessing methods to obtain optimized models. Ultimately, the performance improvement of the combination model (Model 4) compared to Model 3 was then confirmed.

## RESULTS AND DISCUSSION

### The change in meat quality of pork loin during vacuum storage

**Figure 1:**
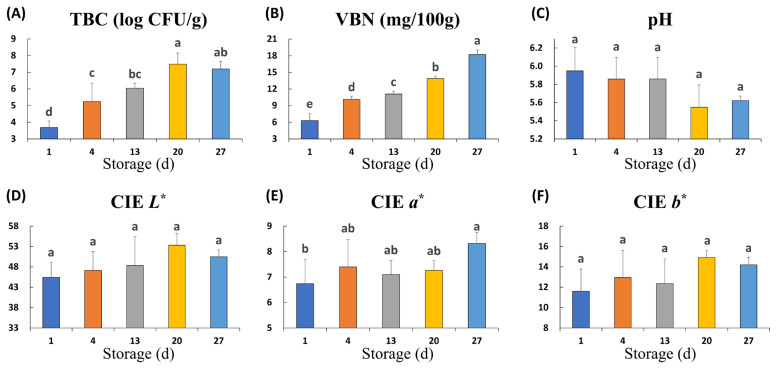
Physicochemical and microbial properties change of pork loin during storage. The letters at the top of the bar chart represent values indicating significant difference between the values in the different storage days (p<0.05). (A) Total bacterial count (TBC), (B) volatile basic nitrogen (VBN), (C) pH, (D) CIE L*, (E) CIE a*, and (F) CIE b*.

The results of the microbiological and physicochemical characteristics of the pork loin are presented in Figure 1. Both the TBC and VBN exhibited a significant increase during storage (p<0.05). As mentioned earlier, TBC represents the microbial count in meat, and its value increases during storage due to the abundant nutrients in the meat [1]. VBN increases during storage because enzymes secreted by microorganisms break down muscle proteins into amino acids, leading to an increase in volatile basic nitrogen [7]. TBC and VBN are widely used indicators for determining the freshness or spoilage of meat. Meat is considered fresh if the TBC is below 7.0 log CFU/g and the VBN is below 15 mg/100 g [32,33]. Accordingly, pork meat stored before day 20 was considered safe for consumption. The pH showed a decreasing trend up to 20 days of storage; however, there were no significant differences. The L* value showed no significant difference with storage days, while the a* value exhibited a significant increase on day 27 compared to day 1 (p<0.05). This was consistent with other studies that stored pork loin after vacuum packaging [34,35]. Lastly, the b* value did not show a significant difference with storage days.

### The change in metabolites of pork loin during vacuum storage

**Figure 2:**
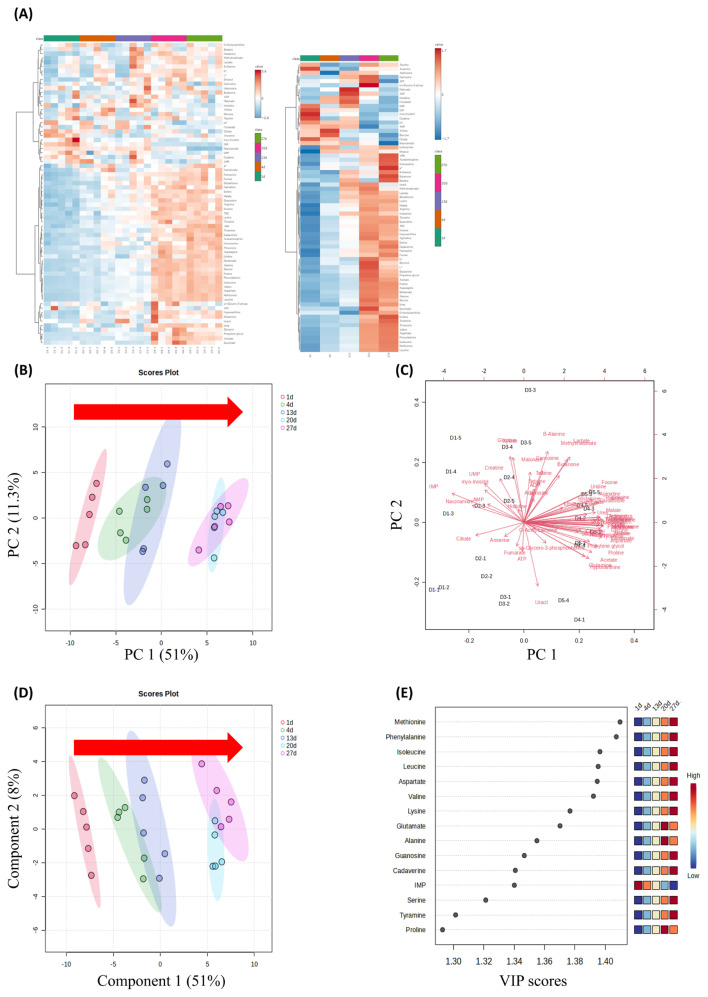
Change of metabolites of refrigerated pork during storage. (A) Hierarchical clustering Heatmap, (B) core plot by principal component analysis (PCA), (C) Biplot by PCA, (D) score plot by partial least squares-discriminant analysis (PLS-DA), and (E) variable importance in projection (VIP) scores by PLS-DA.

The NMR analysis of pork loin revealed a total of 64 metabolites (Supplementary Table S1). According to the heatmap results, compared to day 1, 36 metabolites showed a significant increase on day 27, 4 metabolites showed a significant decrease, and 24 metabolites showed no significant trend (Figure 2A). The PCA results indicated that PC1 and PC2 accounted for 51% and 11.3% of the variability, respectively. In this case, the samples were categorized into three groups based on the metabolites (Group 1: day 1; Group 2: days 4 and 13; Group3: days 20 and 27) (Figure 2B). With the increase in storage days, the PC1 values increased. Moreover, the content of metabolites such as creatine and histidine increased with storage days, shifting to the right from the center of the biplot (Figure 2C). Conversely, the composition of metabolites, including valine, isoleucine, and glycine decreased with storage days, moving to the left from the center (Figure 2C). The results of PLS-DA also revealed three groups, similar to the PCA results. It exhibited a pattern moving to the right with storage duration as the basis, particularly along component 1 (explaining 51% of the variance) (Figure 2D). The trends of these metabolites during spoilage were akin to those in the study by Yu et al [36]. Lastly, based on the VIP score results for each metabolite, it was evident that methionine, phenylalanine, leucine, and aspartate significantly influenced the differentiation of samples according to storage days (Figure 2E).

### Prediction of pork loin qualities using metabolites

**Figure 3:**
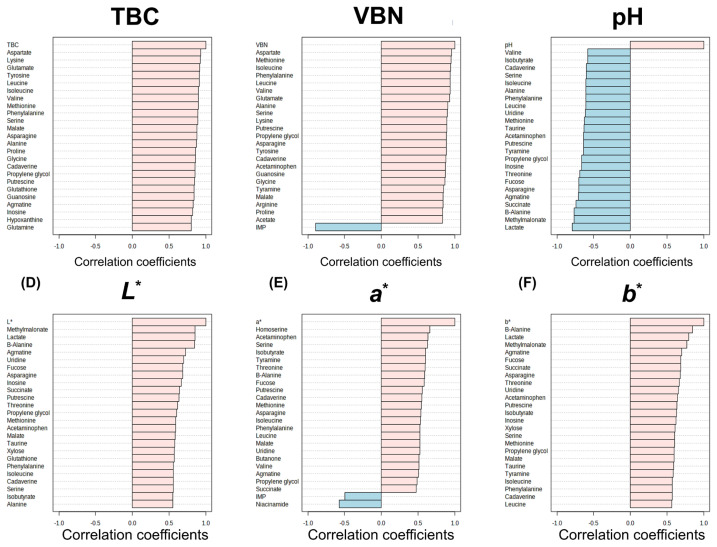
A bar graph showing the correlation coefficients of the top 24 metabolites with quality traits of refrigerated pork. (A) Total bacterial count (TBC), (B) volatile basic nitrogen (VBN), (C) pH, (D) L*, (E) a*, and (F) b*.

Pearson correlation coefficients between the 64 metabolites and meat quality traits (TBC, VBN, pH, and meat color) were conducted to understand which meat qualities show high correlations with metabolites changes in meat during storage (Figure 3). TBC (0.7983<*r*<0.9107) and VBN (0.7942 <*r*<0.9242) showed higher correlations coefficients with metabolites than other meat qualities (0.4444<*r*<0.8450).

**Figure 4:**
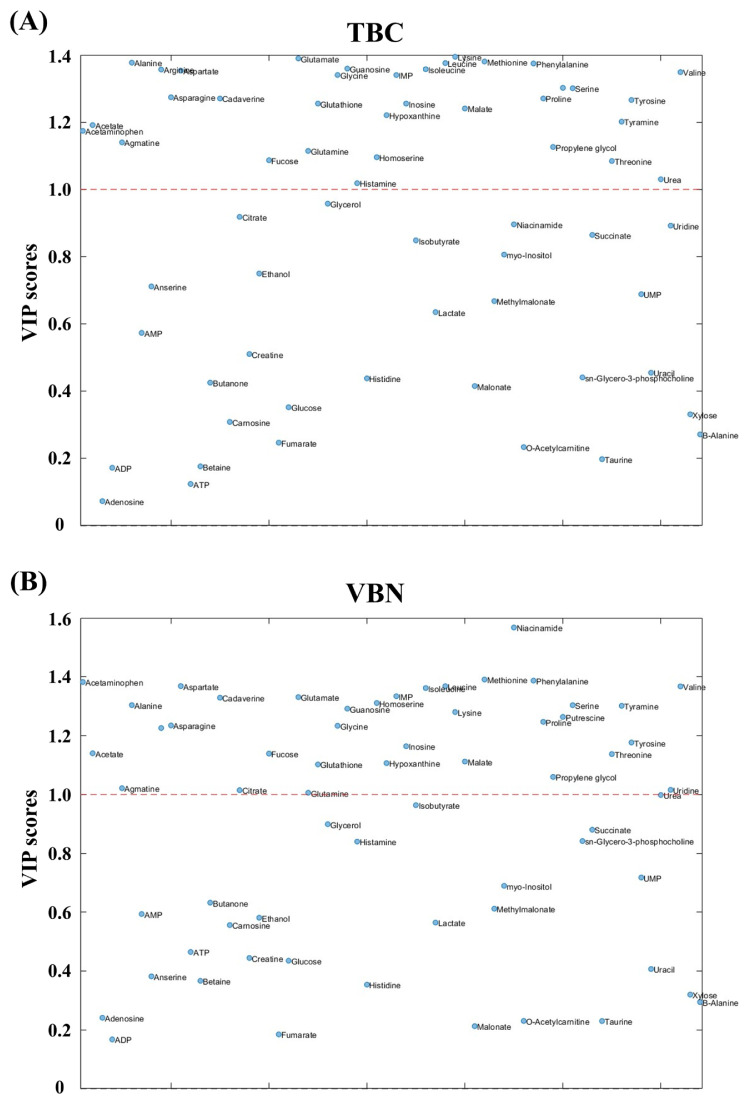
Variable importance in projection (VIP) scores plot metabolites with total bacterial count (TBC) and volatile basic nitrogen (VBN). (A) VIP scores for TBC, and (B) VIP scores for VBN. The data were pre-processed by auto-scaling.

Subsequently, the performance of PLSR models for TBC, VBN, pH, and meat color using the metabolites was compared to determine which meat qualities could effectively be predicted by metabolites (Table 1). The results showed that the R2 values for TBC and VBN were 0.8136 and 0.9364, respectively, which were higher than those for other meat qualities. This indicates that metabolites could effectively predict TBC and VBN. Therefore, TBC and VBN were considered as the freshness indicators of pork meat during storage, which showed a high relation with metabolites and could be predictable by metabolites. This result aligns with the findings in another study, which mentioned TBC and VBN as crucial components in the freshness indices [7]. Moreover, to assess which metabolites had an impact on the prediction of TBC and VBN, VIP scores were analyzed (Figure 4). The results revealed that there were 34 and 35 metabolites for TBC and VBN, respectively, with VIP scores greater than 1 (Table 2), and these metabolites mostly showed a high correlation with TBC and VBN in Figure 3.

### Linear regression for predicting pork loin qualities using metabolites

Table 3 presents the equations obtained through linear regression in SPSS using the identified metabolites for TBC and VBN (Table 2). The profit models for TBC and VBN were selected according to the previous criteria (VIF<10, CI<15) [29]. As a result, according to the stepwise method, a model with lysine and malate could predict a TBC value with an R^2^ of 0.886. Similarly, a model utilizing methionine and histidine was produced for predicting VBN with an R^2^ of 0.909. The equations for TBC and VBN were as below:


$$\begin{array}{l} \text{TBC}=2.316+0.120\times (\text{Lysine})+0.288\times (\text{Malate})\\ \text{VBN}=9.727+0.614\times (\text{Methionine})-0.923\times (\text{Niacinamide}) \end{array}$$

In the equation for TBC, the coefficient for malate was approximately 2.4 times higher than that for lysine. In the case of VBN, niacinamide had a coefficient about 1.5 times higher than methionine. This suggests that in both formulas, malate and niacinamide have a significant impact on the quality indicators compared to other metabolites.

Lysine and methionine are known to be involved in glutathione metabolism, potentially safeguarding cellular components against reactive substances generated during stress-induced oxidation [25]. Both metabolites showed an increase during the storage period (Supplementary Table S1); thus, this phenomenon could be interpreted as preventing the impact of microbial byproducts on meat during storage. Moreover, the increasing trend in storage days according to the primary metabolites aligns with the findings of a previous study [37]. Malate is known as an intermediate in the tricarboxylic acid (TCA) cycle, which is a crucial part of the energy metabolism process that occurs in the mitochondria [38]. Furthermore, it has been documented that malate can be generated through microbial fermentation, including organisms such as *Escherichia coli*, *Rhizopus oryzae*, and others [39]. Finally, niacinamide is a type of vitamin B_3_ that helps prevent diseases such as pellagra and dermatitis. This can be converted to NADH and NADPH, which play important roles in many biochemical reactions [40]. In our experimental results, niacinamide showed a decreasing trend according to storage days. According to Muroya et al [41], niacinamide showed a gradual increase in the *Longissimus thoracis* (LT) muscle of Japanese Brown cattle depending on the 14 days of storage, while NAD^+^ exhibited a decreasing trend. NAD^+^ is typically utilized in energy metabolism under conditions with oxygen, such as the TCA cycle, and its consumption results in the conversion to niacinamide [42,43]. However, in our study, niacinamide tended to decrease, possibly due to limited oxygen entry caused by vacuum packaging, preventing the proper breakdown of NAD^+^.

### Prediction of selected metabolites and freshness index with HSI spectral data

**Figure 5:**
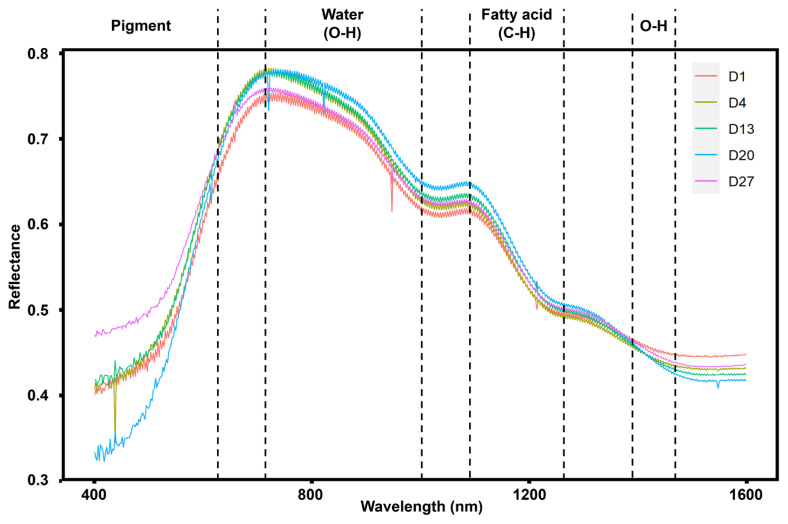
The reflectance of meat samples in different wavelengths. The range of the wavelength is from 400 to 1,600 nm. The total number of wavelengths is 537.

Figure 5 represents the HSI spectral data showing the average reflectance values for samples at different wavelengths. To optimize the model, PLSR modeling was conducted for the four selected metabolites (lysine, malate, methionine, and niacinamide) with different preprocessing methods such as SNV, normalization, and MSC (Table 4). The modeling results indicated that for lysine and malate, the prediction coefficient of determination (R^2^_P_) was best when using raw HSI spectral data and normalized HSI spectral data, with values of 0.8108 and 0.6862, respectively. Next, for methionine and niacinamide, the model performance was highest when using the SNV preprocessing method for HSI spectral data, with values of 0.8130 and 0.7327, respectively. SNV is known to remove the noise effects arising from the size of solid particles, surface scattering, and variations in the light range on the diffuse reflectance spectrum [44]. As meat exhibits a highly uneven surface compared to other food types, the SNV method could effectively reduce these variations and enhance the model’s coefficient.

**Figure 6:**
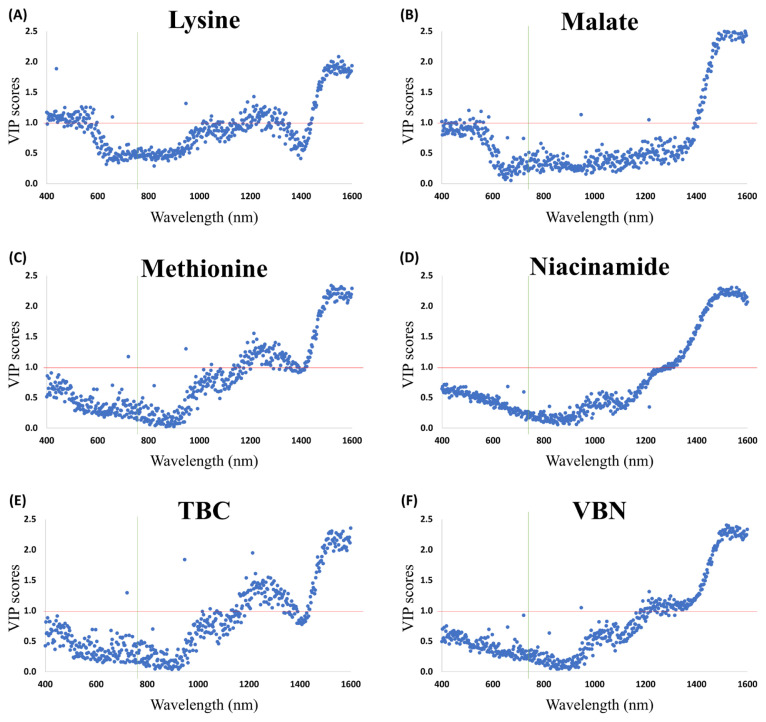
Variable importance in projection (VIP) score plots of selected metabolites, total bacterial count (TBC), and volatile basic nitrogen (VBN) with HSI spectral data by partial least squares regression (PLSR). The red line shows the number 1 of the VIP score and the green line represents the 780 nm of wavelength which is the boundary of visible and near-infrared regions of the wavelengths. (A) Lysine, (B) Malate, (C) Methionine, (D) Niacinamide, (E) TBC, and (F) VBN.

Furthermore, for each metabolite, the wavelengths with high VIP scores (>1.0) were identified (Figure 6). Lysine and malate, associated with TBC, consistently exhibited high VIP scores (>1.0) at various wavelengths, including 400 to 585 nm in the visible light range and 947, 1,214, and 1,441 to 1,599 nm in the NIR range (Figure 6A and 6B). Methionine and niacinamide, which represent the value of VBN, also exhibited high VIP scores (>1.0) at 1,271 to 1,599 nm in the NIR range (Figure 6C and 6D). The optimal model for TBC (R^2^_p_ = 0.7220) and VBN (R^2^_p_ = 0.8392) was attained by preprocessing the HSI spectral data with SNV (Table 4). Identifying the wavelengths with VIP scores greater than 1 revealed that for TBC, these wavelengths were 721 nm in the visible light range and 947 nm, as well as 1,047 to 1,599 nm in the NIR range (Figure 6E). For VBN, these wavelengths were not present in the visible light range and were observed at 947 nm, as well as 1,180 to 1,599 nm in the NIR range (Figure 6F). Finally, when examining the common wavelengths with a VIP score greater than 1 for freshness indicators and metabolites, TBC with lysine and malate was observed at 947, 1,214, and 1,441 to 1,599 nm, while VBN with methionine and niacinamide appeared around 1,271 to 1,599 nm.

Wavelengths near 721 and 947 nm are recognized to correspond to the water absorption bands related to O-H stretching and bending overtones [45]. Additionally, wavelengths within the range of 1,100 to 1,214 nm are associated with the second overtone of C-H stretching vibration in the radicals C-H, C-H_2_, and C-H_3_ of triglycerides [46]. Furthermore, wavelengths ranging from 1,396 to 1,599 nm are acknowledged as the first overtone of the O-H and N-H stretching modes of water-bonded groups [6]. However, in another study that proposed the absorption spectra of protein and amino acids to be under 300 nm, expanding the HSI spectral range to shorter wavelengths might have yielded more improved results [47].

### Making combination models to improve model performance for predicting TBC and VBN values

**Figure 7:**
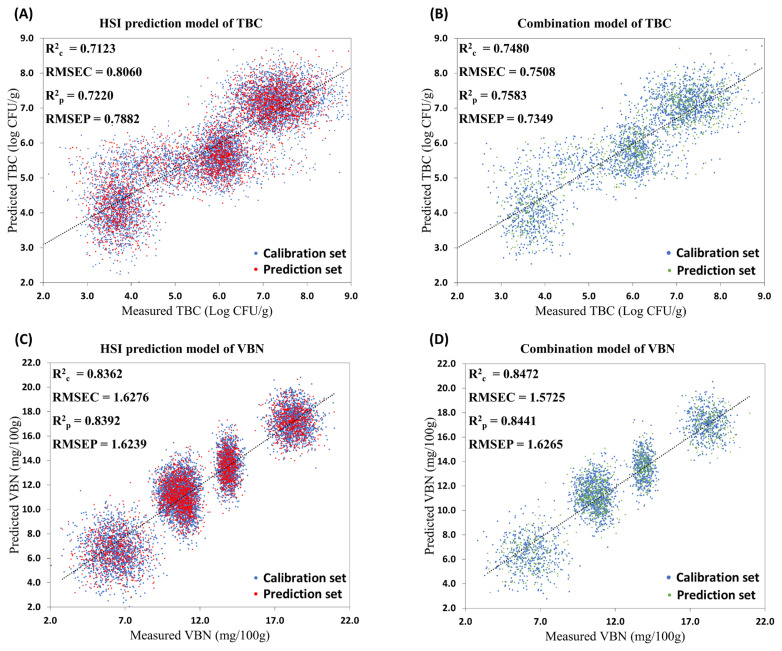
Scatterplot of total bacterial count (TBC) and volatile basic nitrogen (VBN) prediction results. The dashed line represents the trendline of the calibration set. (A) Model 3 for TBC made with standard normal variate (SNV) preprocessed hyperspectral imaging (HSI) data, (B) Model 4 for TBC made with SNV preprocessed HSI, lysine, and malate, (C) Model 3 for VBN made with SNV preprocessed HSI data, and (D) Model 4 for VBN made with SNV preprocessed HSI, methionine, and niacinamide.

To improve the performance of the TBC and VBN prediction models using HSI and metabolites, a combination of HSI spectral data and selected metabolites data (lysine, malate, methionine, and niacinamide) were employed. The models were reconstructed using HSI data and selected metabolites (lysine and malate for TBC, methionine, and niacinamide for VBN) as X variables. The metabolites dataset consisted of the predicted metabolites obtained through Model 2, and the HSI spectral data was the prediction set used for making both Model 2 and 3 (n = 3,000). The results of this combination model with various preprocessing methods are shown in Supplementary Table S2. Among them, the best model for each freshness indicator was obtained with SNV preprocessing. The predictive performance of the combination model (R^2^_p_) for TBC increased by 0.0363, and for VBN, it increased by 0.0049 compared to the original freshness models (Figure 7).

These results may come from the microbial metabolism in meat during storage, as the selected freshness indicators in this study (TBC and VBN) have a highly significant relationship with microorganisms. As the meat spoils, TBC in the meat increases, leading to the generation of VBN compounds such as dimethylamine, and ammonia. A previous study indicated that peaks in HSI spectral data around 930 and 1,121 nm were related to *Enterobacteriaceae*, while peaks around 1,138 to 1,200, 1,450, and approximately 1,525 nm were related to *Pseudomonas* spp., both known to impact meat spoilage [48]. An increase in microorganisms could result in elevated oxidative stress on pork loin [49]. Among the selected metabolites, lysine and methionine are related to glutathione metabolism, which could contribute to the oxidation resistance of pork loin. Malate and niacinamide are associated with the TCA cycle and energy metabolism of microorganisms. Therefore, the interconnected relationship between HSI spectral data, TBC, VBN, and four selected metabolites might work effectively in creating a performative combined modeling system. However, we have only suggested the possibility that specific microorganisms may have influenced certain peaks in the spectral data and consequently the spoilage of the meat. To confidently establish a direct relationship among metabolites, freshness indicators, and microorganisms, both qualitative and quantitative analyses of the types of microorganisms present in pork loin are necessary.

## CONCLUSION

TBC and VBN exhibited a high correlation with changes in metabolites and were identified as freshness indicators through PLSR modeling. Lysine and malate for TBC, and methionine and niacinamide for VBN were well explained as each freshness indicator. These were selected using linear regression models with a stepwise method. Ultimately, the predictive performance (R^2^_p_) of the combination model established with selected metabolites and HSI data as X variables increased by 0.0363 for TBC and 0.0049 for VBN compared to model 3 which contained only HSI data in X variables. Therefore, the conclusion is drawn that combining HSI spectral data with correlated metabolite data improves the prediction of pork freshness compared to using HSI spectral data alone, as a non-destructive method. While further research under various conditions of pork loin is necessary for practical application in the meat industry, demonstrating the potential use of specific metabolites in developing HSI spectral models for meat spoilage is worthwhile.

## Figures and Tables

**Table 1:** The performance of Model 1

Trait	LVs	RMSEC	RMSECV	RMSEP	R^2^_C_	R^2^_CV_	R^2^_P_
TBC	2	0.6605	0.6617	0.6604	0.8117	0.8109	0.8136
VBN	2	1.0048	1.0066	1.0219	0.9372	0.9370	0.9364
pH	2	0.2215	0.2220	0.2281	0.3140	0.3109	0.3007
L*	2	4.4389	4.4517	4.4019	0.2703	0.2661	0.2892
a*	2	0.7440	0.7455	0.7365	0.3137	0.3109	0.3224
b*	2	1.9186	1.9239	1.9405	0.2709	0.2669	0.2640

LVs, latent variables; RMSE, root mean square error; R^2^, coefficient of determination; C, calibration; CV, cross-validation; P, prediction; TBC, total bacterial count; VBN, volatile basic nitrogen.

**Table 2:** The selected metabolites highly correlated with total bacterial count (TBC) and volatile basic nitrogen (VBN)

Quality	Metabolites (VIP score >1.0)	Total number
TBC	Acetaminophen, acetate, agmatine, alanine, arginine, asparagine, aspartate, cadaverine, fucose, glutamate, glutamine, glutathione, glycine, guanosine, histamine, homoserine, hypoxanthine, inosine monophosphate, inosine, isoleucine, leucine, lysine, malate, methionine, phenylalanine, proline, propylene glycol, putrescine, serine, threonine, tyramine, tyrosine, urea, valine	34
VBN	Acetaminophen, acetate, agmatine, alanine, arginine, asparagine, aspartate, cadaverine, citrate, fucose, glutamate, glutamine, glutathione, glycine, guanosine, homoserine, hypoxanthine, inosine monophosphate, inosine, isoleucine, leucine, lysine, malate, methionine, niacinamide, phenylalanine, proline, propylene glycol, putrescine, serine, threonine, tyramine, tyrosine, uridine, valine	35

VIP, variable importance in projection.

**Table 3:** The linear regression results of total bacterial count (TBC) and volatile basic nitrogen (VBN) with metabolites

Y	R^2^	Metabolites	Coefficient	VIF	CI
TBC	0.886	(constant)	2.316		1.000
		Lysine	0.120	3.417	5.150
		Malate	0.288	3.417	12.140
VBN	0.909	(constant)	9.727		1.000
		Methionine	0.614	1.972	2.739
		Niacinamide	−0.923	1.972	10.793

R^2^, coefficient of determination; VIF, variance inflation factor; CI, condition index.

**Table 4:** The performance of Model 2 and Model 3 in different preprocessing methods

Objective	Preprocessing	LVs	RMSEC	RMSECV	RMSEP	R^2^_C_	R^2^_CV_	R^2^_P_
Model 2								
Lysine	RAW	3	2.9318	3.0166	3.0595	0.8252	0.8150	0.8108
	SNV	3	2.9993	3.1353	3.1540	0.8171	0.8001	0.7991
	Normalize	3	3.0904	3.1766	3.2034	0.8058	0.7948	0.7927
	MSC (Mean)	2	3.1978	3.2417	3.2549	0.7921	0.7864	0.7859
Malate	RAW	2	1.2749	1.2914	1.2796	0.6723	0.6638	0.6663
	SNV	2	1.3394	1.3572	1.3105	0.6383	0.6287	0.6498
	Normalize	3	1.2306	1.2717	1.2410	0.6947	0.6741	0.6862
	MSC (Mean)	2	1.4413	1.4626	1.4223	0.5812	0.5687	0.5876
Methionine	RAW	3	2.4621	2.5435	2.5448	0.7258	0.7074	0.7124
	SNV	2	2.0348	2.0586	2.0525	0.8127	0.8083	0.8130
	Normalize	3	2.3462	2.4203	2.4273	0.7510	0.7350	0.7384
	MSC (Mean)	2	2.5864	2.6226	2.6275	0.6974	0.6889	0.6936
Niacinamide	RAW	3	0.9125	0.9450	0.9456	0.5737	0.5433	0.5595
	SNV	2	0.7338	0.7422	0.7368	0.7243	0.7180	0.7327
	Normalize	4	0.7482	0.8001	0.7928	0.7135	0.6729	0.6904
	MSC (Mean)	2	0.9711	0.9852	0.9833	0.5172	0.5032	0.5241
Model 3								
TBC	RAW	3	0.8031	0.8282	0.8060	0.7144	0.6963	0.7094
	SNV	2	0.8060	0.8160	0.7882	0.7123	0.7051	0.7220
	Normalize	3	0.8028	0.8269	0.8011	0.7146	0.6972	0.7129
	MSC (Mean)	2	0.8744	0.8866	0.8618	0.6614	0.6519	0.6677
VBN	RAW	3	2.1464	2.2194	2.1713	0.7151	0.6955	0.7125
	SNV	2	1.6276	1.6478	1.6239	0.8362	0.8321	0.8392
	Normalize	3	2.0244	2.0935	2.0509	0.7466	0.7290	0.7436
	MSC (Mean)	2	2.1979	2.2304	2.1939	0.7013	0.6924	0.7067

LVs, latent variables; RMSE, root mean square error; R^2^, coefficient of determination; C, calibration; CV, cross-validation; P, prediction; SNV, standard normal variate; MSC, multiplicative scatter correction; TBC, total bacterial count; VBN, volatile basic nitrogen.

## SUPPLEMENTARY MATERIAL

**Supplementary Table S1**. The metabolites concentration results of nuclear magnetic resonance of pork loin during storage

**Supplementary Table S2**. PLSR combination model for predicting the total bacterial count (TBC) and volatile basic nitrogen (VBN) (Model 4)

**Supplementary Figure S1**. A schematic diagram of the sample preparation process. Both sides of the pork loin were utilized.

**Supplementary Figure S2**. The graphical abstract of the entire experiment process.
