# Supplementary material for: Combination model for freshness prediction of pork using VIS/NIR hyperspectral imaging with chemometrics
Source: Anim Biosci. 2024 Aug 26;38(1):142–56. doi: 10.5713/ab.24.0255 (PMC11725733; doi:10.5713/ab.24.0255)
Supplement: Supplementary file 1 [file ab-24-0255-Supplementary-Table-1.pdf]

**Supplementary data**

**Table S1.** The metabolites concentration results of nuclear magnetic resonance of pork loin during storage.

| Metabolites (mg/dL) | Days | | | | | SEM |
|---|---|---|---|---|---|---|
| | 1 | 4 | 13 | 20 | 27 | |
| Acetaminophen | 0.260$^{D}$ | 0.734$^{BC}$ | 0.616$^{CD}$ | 1.106$^{B}$ | 1.495$^{A}$ | 0.0915 |
| Acetate | 1.598$^{B}$ | 2.305$^{B}$ | 3.755$^{B}$ | 11.248$^{A}$ | 9.756$^{A}$ | 0.8775 |
| Adenosine | 6.511 | 3.692 | 6.522 | 6.373 | 5.725 | 1.0899 |
| ADP | 4.318 | 4.312 | 4.794 | 4.575 | 4.350 | 0.3185 |
| Agmatine | 1.874$^{C}$ | 4.238$^{BC}$ | 5.837$^{AB}$ | 9.024$^{A}$ | 7.566$^{AB}$ | 0.8694 |
| Alanine | 10.337$^{D}$ | 14.277$^{C}$ | 18.104$^{B}$ | 30.192$^{A}$ | 28.367$^{A}$ | 0.7249 |
| AMP | 3.556 | 3.654 | 3.585 | 2.680 | 3.116 | 0.2886 |
| Anserine | 18.867$^{A}$ | 11.041$^{BC}$ | 8.918$^{C}$ | 13.861$^{B}$ | 13.815$^{B}$ | 1.0300 |
| Arginine | 3.715$^{C}$ | 11.446$^{B}$ | 12.004$^{B}$ | 18.109$^{A}$ | 16.590$^{A}$ | 0.8635 |
| Asparagine | 1.008$^{B}$ | 2.172$^{B}$ | 3.052$^{B}$ | 8.370$^{A}$ | 7.874$^{A}$ | 0.5089 |
| Aspartate | 0.657$^{C}$ | 2.195$^{B}$ | 3.321$^{B}$ | 7.391$^{A}$ | 8.110$^{A}$ | 0.2804 |
| ATP | 6.641 | 6.777 | 6.972 | 7.483 | 6.030 | 0.6980 |
| Betaine | 3.196 | 3.069 | 3.830 | 3.112 | 4.119 | 0.6899 |
| Butanone | 0.483 | 0.485 | 0.590 | 0.539 | 0.676 | 0.0676 |
| Cadaverine | 0.715$^{D}$ | 1.136$^{CD}$ | 1.402$^{BC}$ | 1.835$^{AB}$ | 2.122$^{A}$ | 0.1057 |
| Carnosine | 332.255 | 333.300 | 352.190 | 361.113 | 327.628 | 13.0450 |
| Citrate | 1.889$^{A}$ | 1.645$^{AB}$ | 1.247$^{BC}$ | 1.432$^{ABC}$ | 1.132$^{C}$ | 0.1113 |
| Creatine | 227.771 | 195.700 | 213.928 | 194.693 | 205.048 | 9.3052 |
| Ethanol | 0.687$^{B}$ | 2.669$^{A}$ | 2.612$^{A}$ | 2.585$^{A}$ | 2.311$^{AB}$ | 0.3994 |
| Fucose | 1.454$^{C}$ | 2.878$^{BC}$ | 3.345$^{AB}$ | 3.801$^{AB}$ | 4.474$^{A}$ | 0.3558 |
| Fumarate | 0.741 | 0.858 | 0.914 | 0.777 | 0.712 | 0.0875 |
| Glucose | 28.530 | 33.395 | 24.556 | 25.814 | 19.169 | 5.2678 |
| Glutamate | 3.960$^{D}$ | 10.843$^{C}$ | 14.430$^{B}$ | 30.820$^{A}$ | 29.133$^{A}$ | 0.7903 |

| | | | | | | |
|---|---|---|---|---|---|---|
| Glutamine | 10.340$^{C}$ | 12.387$^{BC}$ | 13.346$^{BC}$ | 17.253$^{A}$ | 15.507$^{AB}$ | 0.8799 |
| Glutathione | 1.779$^{B}$ | 3.480$^{B}$ | 7.129$^{A}$ | 7.825$^{A}$ | 7.606$^{A}$ | 0.5563 |
| Glycerol | 11.411$^{C}$ | 15.341$^{BC}$ | 14.435$^{BC}$ | 21.628$^{A}$ | 17.469$^{AB}$ | 1.4052 |
| Glycine | 6.317$^{C}$ | 7.767$^{C}$ | 10.893$^{B}$ | 17.158$^{A}$ | 14.965$^{A}$ | 0.5550 |
| Guanosine | 0.382$^{C}$ | 0.776$^{B}$ | 0.990$^{B}$ | 1.387$^{A}$ | 1.407$^{A}$ | 0.0654 |
| Histamine | 0.177$^{B}$ | 0.264$^{AB}$ | 0.310$^{A}$ | 0.326$^{A}$ | 0.302$^{A}$ | 0.0229 |
| Histidine | 0.431$^{B}$ | 0.586$^{AB}$ | 0.634$^{A}$ | 0.516$^{AB}$ | 0.420$^{B}$ | 0.0461 |
| Homoserine | 5.324$^{C}$ | 10.318$^{B}$ | 9.776$^{B}$ | 11.978$^{AB}$ | 15.592$^{A}$ | 0.9575 |
| Hypoxanthine | 7.404$^{C}$ | 13.149$^{BC}$ | 18.238$^{AB}$ | 26.918$^{A}$ | 24.348$^{A}$ | 2.0607 |
| Inosine monophosphate | 102.039$^{A}$ | 66.954$^{B}$ | 65.181$^{B}$ | 33.972$^{C}$ | 27.578$^{C}$ | 4.2554 |
| Inosine | 25.525$^{D}$ | 36.695$^{C}$ | 40.713$^{BC}$ | 52.166$^{A}$ | 49.972$^{AB}$ | 2.5323 |
| Isobutyrate | 0.079$^{C}$ | 0.351$^{A}$ | 0.182$^{BC}$ | 0.301$^{AB}$ | 0.349$^{A}$ | 0.0306 |
| Isoleucine | 2.150$^{C}$ | 5.402$^{B}$ | 7.338$^{B}$ | 14.399$^{A}$ | 15.699$^{A}$ | 0.5386 |
| Lactate | 406.541 | 423.068 | 464.714 | 484.487 | 472.651 | 25.6375 |
| Leucine | 4.056$^{C}$ | 10.862$^{B}$ | 13.772$^{B}$ | 25.091$^{A}$ | 26.798$^{A}$ | 0.9316 |
| Lysine | 4.275$^{D}$ | 8.122$^{C}$ | 16.491$^{B}$ | 21.058$^{A}$ | 21.171$^{A}$ | 0.6838 |
| Malate | 3.121$^{C}$ | 6.322$^{B}$ | 7.054$^{AB}$ | 8.671$^{A}$ | 8.234$^{AB}$ | 0.5059 |
| Malonate | 4.416 | 4.652 | 5.140 | 4.617 | 4.422 | 0.1768 |
| Methionine | 1.956$^{C}$ | 5.579$^{B}$ | 7.509$^{B}$ | 12.974$^{A}$ | 14.314$^{A}$ | 0.4734 |
| Methylmalonate | 3.244 | 3.300 | 3.917 | 4.099 | 3.728 | 0.2411 |
| myo-Inositol | 7.268$^{A}$ | 4.250$^{AB}$ | 4.664$^{AB}$ | 3.093$^{B}$ | 4.192$^{AB}$ | 0.8240 |
| Niacinamide | 4.196$^{AB}$ | 3.629$^{AB}$ | 4.200$^{A}$ | 3.311$^{B}$ | 0.586$^{C}$ | 0.2027 |
| O-Acetylcarnitine | 1.718 | 1.720 | 1.591 | 1.873 | 1.848 | 0.1971 |
| Phenylalanine | 2.596$^{D}$ | 5.962$^{C}$ | 8.132$^{B}$ | 15.174$^{A}$ | 16.450$^{A}$ | 0.4669 |
| Proline | 3.613$^{B}$ | 4.704$^{B}$ | 5.422$^{B}$ | 10.376$^{A}$ | 10.099$^{A}$ | 0.4610 |
| Propylene glycol | 0.038$^{C}$ | 0.106$^{C}$ | 0.188$^{BC}$ | 0.581$^{A}$ | 0.447$^{AB}$ | 0.0623 |
| Putrescine | 0.703$^{D}$ | 1.366$^{C}$ | 1.563$^{BC}$ | 1.846$^{AB}$ | 1.960$^{A}$ | 0.0936 |
| Serine | 4.286$^{C}$ | 10.646$^{B}$ | 12.215$^{B}$ | 17.572$^{A}$ | 19.211$^{A}$ | 1.0930 |

| | | | | | | |
|---|---|---|---|---|---|---|
| sn-Glycero-3-phosphocholine | 22.354$^{AB}$ | 21.135$^{B}$ | 21.813$^{AB}$ | 27.337$^{A}$ | 21.724$^{B}$ | 1.3260 |
| Succinate | 1.631$^{BC}$ | 1.699$^{BC}$ | 0.740$^{C}$ | 6.099$^{A}$ | 4.035$^{AB}$ | 0.6401 |
| Taurine | 16.782 | 12.352 | 13.094 | 17.042 | 15.863 | 2.1780 |
| Threonine | 2.352$^{B}$ | 4.428$^{B}$ | 3.840$^{B}$ | 12.498$^{A}$ | 13.719$^{A}$ | 1.3436 |
| Tyramine | 1.120$^{B}$ | 1.723$^{B}$ | 2.174$^{B}$ | 4.814$^{A}$ | 5.840$^{A}$ | 0.3649 |
| Tyrosine | 1.079$^{C}$ | 3.814$^{BC}$ | 5.921$^{AB}$ | 8.560$^{A}$ | 8.489$^{A}$ | 0.7246 |
| UMP | 1.518 | 1.159 | 1.466 | 1.076 | 1.057 | 0.1137 |
| Uracil | 0.141 | 0.252 | 0.397 | 0.406 | 0.261 | 0.1097 |
| Urea | 1.734$^{C}$ | 1.615$^{C}$ | 2.114$^{BC}$ | 3.006$^{A}$ | 2.926$^{AB}$ | 0.2100 |
| Uridine | 0.673$^{B}$ | 0.744$^{B}$ | 0.793$^{B}$ | 1.130$^{AB}$ | 1.356$^{A}$ | 0.1183 |
| Valine | 2.646$^{C}$ | 5.522$^{B}$ | 7.845$^{B}$ | 16.482$^{A}$ | 17.903$^{A}$ | 0.5492 |
| Xylose | 18.089 | 22.463 | 14.460 | 13.127 | 12.914 | 4.5622 |
| β-Alanine | 2.954 | 2.794 | 3.493 | 3.348 | 3.700 | 0.5664 |

SEM, standard error of the least square mean (n=25).

$^{A\text{-}D}$Different superscripts indicate significant differences among storage days ($p<0.05$).
