# Supplementary material for: Combination model for freshness prediction of pork using VIS/NIR hyperspectral imaging with chemometrics
Source: Anim Biosci. 2024 Aug 26;38(1):142–56. doi: 10.5713/ab.24.0255 (PMC11725733; doi:10.5713/ab.24.0255)
Supplement: Supplementary file 2 [file ab-24-0255-Supplementary-Table-2.pdf]

Table S2. PLSR combination model for predicting the total bacterial count (TBC) and volatile basic nitrogen (VBN) (Model 4).

Objective	Preprocessing	LVs	RMSEC	RMSECV	RMSEP	R^2_C	R^2_{CV}	R^2_P
TBC	RAW	2	0.7939	0.8263	0.8100	0.7183	0.6949	0.7058
	SNV	2	0.7508	0.7777	0.7349	0.7480	0.7299	0.7583
	Normalize	3	0.7425	0.8158	0.7808	0.7536	0.7027	0.7267
	MSC (Mean)	2	0.8127	0.8439	0.8144	0.7047	0.6819	0.7025
VBN	RAW	3	1.6802	1.8833	1.9406	0.8256	0.7810	0.7769
	SNV	2	1.5725	1.6301	1.6265	0.8472	0.8360	0.8441
	Normalize	3	1.6903	1.8585	1.8909	0.8235	0.7867	0.7882
	MSC (Mean)	2	1.9959	2.0817	2.1881	0.7539	0.7324	0.7163

LVs, latent variables; RMSE, root mean square error; R^2, coefficient of determination.
SNV, standard normal variate; MSC, multiplicative scatter correction.
C, CV and P represent calibration, cross-validation, and prediction, respectively.
