# Supplementary material for: Combination model for freshness prediction of pork using VIS/NIR hyperspectral imaging with chemometrics
Source: Anim Biosci. 2024 Aug 26;38(1):142–56. doi: 10.5713/ab.24.0255 (PMC11725733; doi:10.5713/ab.24.0255)
Supplement: Supplementary file 3 [file ab-24-0255-Supplementary-Fig-1.pdf]

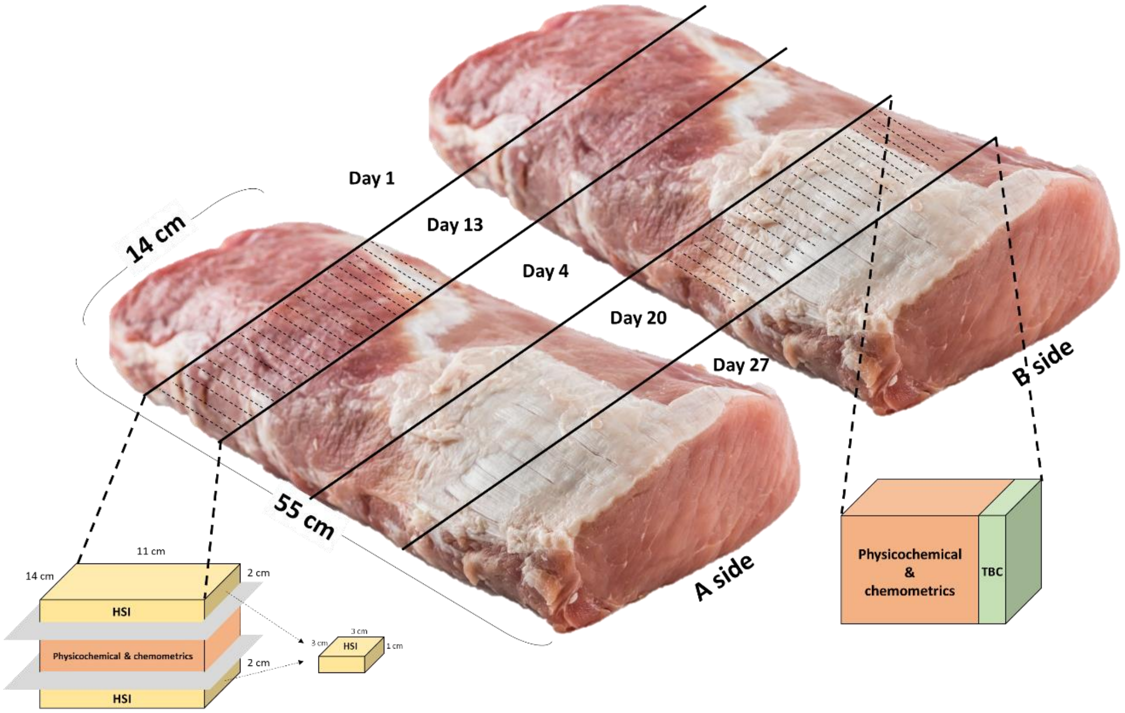

Fig. S1. A schematic diagram of the sample preparation process**.** Both sides of the pork loin were utilized. One side (A side) was used for HSI spectral data collection, physicochemical analysis (pH, meat color, volatile basic nitrogen; VBN), and chemometric analysis. While the other side (B side) was employed for physicochemical and chemometric analyses, and measuring total bacterial count (TBC). Each loin side was divided into five sections and stored on days 1, 4, 13, 20, and 27. For the A-side, as illustrated in the bottom-left diagram, the upper and lower 2 cm of thickness were sectioned into rectangular shapes measuring 3 × 3 × 1 cm (wide × length × height), resulting in a total of 100 standardized samples for HSI spectral data (5 storage days × 5 replication × 4 observation). The remaining portions were used for physicochemical and chemometric analyses. For the B-side, after obtaining the weights used for both TBC and VBN, the remaining portion was utilized for another experiment. The physicochemical and chemometric analyses were conducted on a sample size of 25, representing 5 storage days with 5 replications each.
