# Supplementary material for: Combination model for freshness prediction of pork using VIS/NIR hyperspectral imaging with chemometrics
Source: Anim Biosci. 2024 Aug 26;38(1):142–56. doi: 10.5713/ab.24.0255 (PMC11725733; doi:10.5713/ab.24.0255)
Supplement: Supplementary file 4 [file ab-24-0255-Supplementary-Fig-2.pdf]

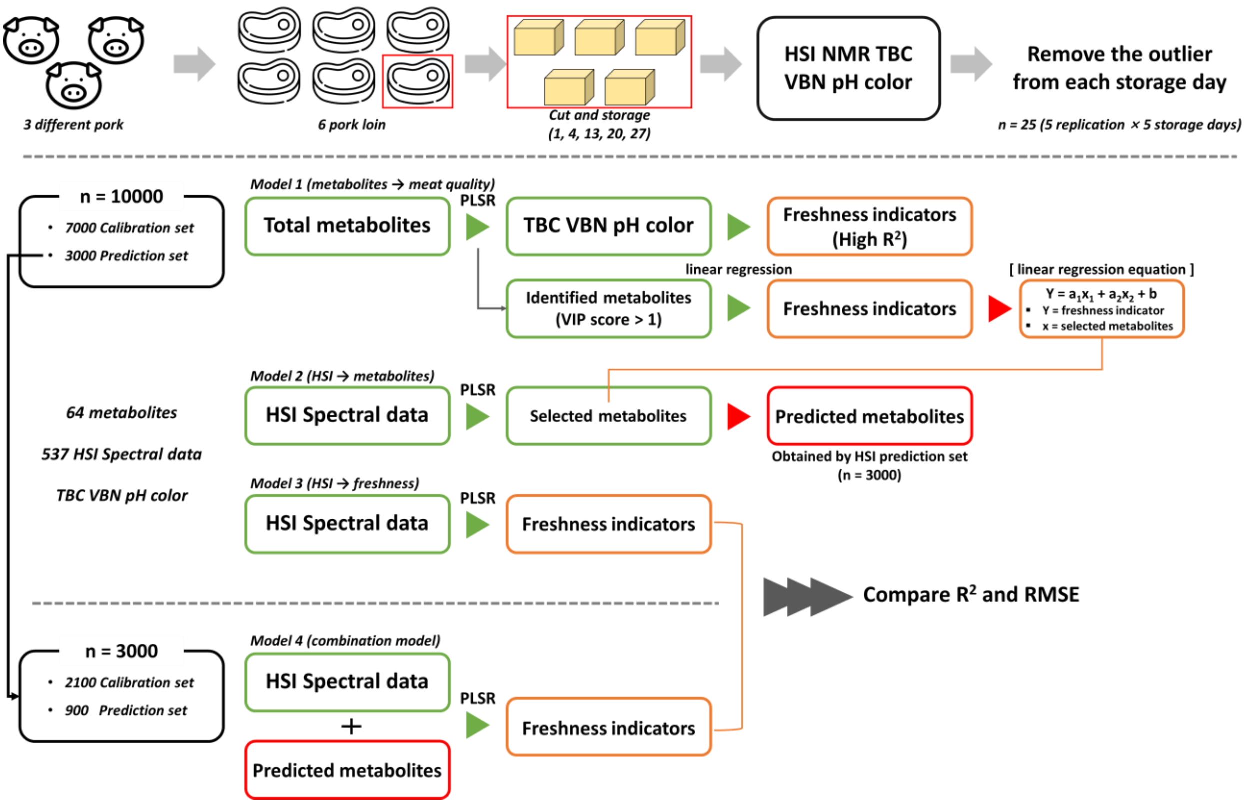

Fig. S2. The graphical abstract of the entire experiment process. Pork loins from three different sows were collected, with both sided being gathered. Each pork loin was subsequently divided into five sub-cuts, representing different time points during storage. Following the measurement of various parameters including HSI data, NMR, TBC, VBN, pH, and meat color, the sample with the highest noise in the NMR analysis at each storage day was considered an outlier and removed from the dataset. Next, modeling process proceeded through several steps. Model 1 involved conducting partial least squares regression (PLSR) on the meat qualities to determine freshness indicators of pork. Linear regression equations for each freshness indicator were made using metabolites with variable importance in projection (VIP) values greater than 1. Model 2 entails creating PLSR models using HSI spectral data for predicting metabolites used in the linear regression equation in Model 1. Model 3 involved making PLSR models predicting freshness indicators using HSI spectral data. Model 4 was a process of reconstructing PLSR models predicting freshness indicators using both HSI spectral data and predicted metabolites dataset. Model 1, 2, and 3 utilized augmented data with n = 10,000 while the Model 4 used its predicted dataset with n = 3,000. The predictive performance of the models is assessed by comparing the coefficient of determination (R^2) and root mean squared error (RMSE).
